# Genome-Wide Diversity, Population Structure and Demographic History of Dromedaries in the Central Desert of Iran

**DOI:** 10.3390/genes11060599

**Published:** 2020-05-29

**Authors:** Morteza Bitaraf Sani, Javad Zare Harofte, Ahmad Bitaraf, Saeid Esmaeilkhanian, Mohammad Hossein Banabazi, Nader Salim, Abbas Teimoori, Ali Shafei Naderi, Mohammad Ali Faghihi, Pamela Anna Burger, Mohammad Silawi, Afsaneh Taghipour Sheshdeh

**Affiliations:** 1 Animal Science Research Department, Yazd Agricultural and Natural Resources Research and Education Center, Agricultural Research, Education & Extension Organization (AREEO), Yazd 8915813155, Iran; javadzare49@gmail.com (J.Z.H.); a_btrf@yahoo.com (A.B.); ashnaderi@gmail.com (A.S.N.); 2 Animal Science Research Institute of Iran, Agricultural Research, Education and Extension Organization (AREEO), Karaj 3146618361, Iran; s.smailkhanian@areeo.ac.ir; 3 Department of Biotechnology, Animal Science Research Institute of IRAN (ASRI), Agricultural Research, Education & Extension Organization (AREEO), Karaj 3146618361, Iran; banabazi@areeo.ac.ir; 4 Organization of Agriculture - Jahad -Yazd, Ministry of Agriculture-Jahad, Yazd 8916713449, Iran; Nader_salim44@yahoo.com (N.S.); teimoori.abbas@yahoo.com (A.T.); 5 Persian BayanGene Research and Training Center, Shiraz, Iran, Center for Therapeutic Innovation and Department of Psychiatry and Behavioral Sciences, University of Miami, Miami, FL 33136, USA; mfaghihi@med.miami.edu; 6 Research Institute of Wildlife Ecology, Vetmeduni Vienna,1160 Vienna, Austria; pamela.burger@vetmeduni.ac.at; 7 Persian BayanGene Research and Training Center, Shiraz 7134767617, Iran; silawimohammad@yahoo.com (M.S.); afsane_t1989@yahoo.com (A.T.S.)

**Keywords:** genotyping-by-sequencing, linkage disequilibrium, effective population Size

## Abstract

The development of camel husbandry for good production in a desert climate is very important, thus we need to understand the genetic basis of camels and give attention to genomic analysis. We assessed genome-wide diversity, linkage disequilibrium (LD), effective population size (Ne) and relatedness in 96 dromedaries originating from five different regions of the central desert of Iran using genotyping-by-sequencing (GBS). A total of 14,522 Single Nucleotide Polymorphisms (SNPs) with an average minor allele frequency (MAF) of 0.19 passed quality control and filtering steps. The average observed heterozygosity in the population was estimated at 0.25 ± 0.03. The mean of LD at distances shorter than 40 kb was low (*r*^2^ = 0.089 ± 0.234). The camels sampled from the central desert of Iran exhibited higher relatedness than Sudanese and lower than Arabian Peninsula dromedaries. Recent Ne of Iran’s camels was estimated to be 89. Predicted Tajima’s D (1.28) suggested a bottleneck or balancing selection in dromedary camels in the central desert of Iran. A general decrease in effective and census population size poses a threat for Iran’s dromedaries. This report is the first SNP calling report on nearly the chromosome level and a first step towards understanding genomic diversity, population structure and demography in Iranian dromedaries.

## 1. Introduction

Camels have a unique morphology and physiology as they are capable of providing vital products in a desert climate, even under harsh conditions. Camelini (one- and two-humped camels) and Lamini (New World camels) are two tribes of the Camelidae family [1]. Camelids influence human endurance and thriving in the peripheral agro-natural zones of (semi-) deserts [2]. Today, camels have gained significance as sustainable livestock species with certain important properties (e.g., immunogenic and milk composition) [3]. Out of around 35 million camel heads of the world (FAO, 2019), the more significant part (95%) are dromedary [4]. There are 138,659 camels in Iran (FAO, 2019), and most of them are dromedaries producing 0.5% of total red meat in Iran [5]. Overuse of land and water resources has expanded the danger of desertification [6]. Over 84% of Iran is arid or semi-arid [7]. Thus, camel husbandry in Iran has a high potential considering changing climatic conditions as well as religious and cultural values [6]. For sustainable development of camel breeding to facilitate protein production in desert climate, we need to give special attention to the conservation and genetic improvement of the species. The main limitations that camel breeding is facing in Iran are the lack of phenotypic records and pedigrees, small herd size and missing connectedness, and genetic evaluations. Previous investigations about genetic diversity and population structure in dromedaries were confined to microsatellite markers, mitochondrial DNA (mtDNA) [8], geographical regions, e.g., Kenya [9], and Pakistan [10], and candidate gene sequencing [11]. Next-generation sequencing platforms have prepared suitable methods for population genetic analyses in local or regional populations/ breeds to investigate effective population size, breed formation, demographic history, selection and genetic drift (interpreted by linkage disequilibrium (LD)) at the whole-genome level [12]. Whole-genome sequencing revealed past demographic events and the evolutionary history of Old World camels [13]. Recently, dromedaries’ genome sequences have been assembled into chromosomes [14,15] and the whole genomes of two Iranian dromedary camels were analyzed. This genomic information could prompt genome-wide association studies (GWAS) and genomic selection in Iranian camel breeding [16], similar to populations from the Arabian Peninsula and Sudan, which showed signatures of selection in regions associated with adaptation to arid environment, dairy traits, energy homeostasis, and chondrogenesis [11]. To understand the genetic makeup and relatedness of Iranian dromedaries and to create the basis for future breeding programs and genomic selection, we assessed the genomic diversity, linkage disequilibrium and population structure of dromedary camels in different regions of the central desert of Iran.

## 2. Materials and Methods

### 2.1. Animal Resources

**Figure 1:**
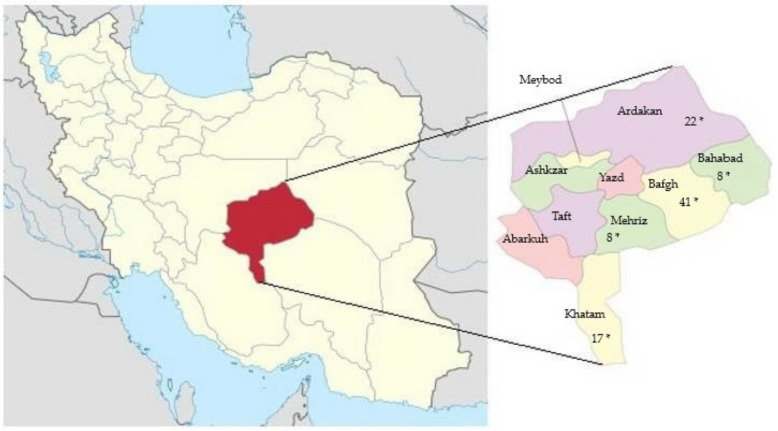
Yazd province, the central desert of Iran. *: samples collected places.

A total of 96 EDTA blood samples of dromedary camels from five regions in Yazd province, Iran, were collected during routine veterinary treatment: (Bafgh (*n* = 41), Bahabad (*n* = 8), Khatam (*n* = 17), Mehriz (*n* = 8) and Saghand (*n* = 22)) (Figure 1).

### 2.2. Genotyping-by-Sequencing (GBS) Data

The blood samples were gathered with EDTA tubes, and genomic DNA was extracted using the modified salt-out method. In this method, optimization includes utilization of separate buffers instead of buffy coat isolation, chloroform for DNA phase isolation and extraction of purified DNA, and sodium acetate for more concentrated DNA [17]. The extracted DNA was evaluated with a Nanodrop Spectrophotometer and 96 samples were genotyped-by-sequencing using two restriction enzymes, EcoR1 and HinF1, and paired-end (150 bp) sequencing on the Illumina HiSeq 2000 platform by Persian Bayangene Research and Training Center (Shiraz, Iran). The adapters were trimmed with bcl2fastq, and the read pairs with low quality were omitted (base Qphred ≤20) by using fastQC.

### 2.3. SNP Calling

The sequence reads were mapped to the dromedary reference genome assembly on chromosome level (GCA_000803125.3; [14]) by using the BWA-MEM algorithm of the Burrows—Wheeler Aligner (BWA; [18]). The PCR duplicates were detected by utilizing Picard tools and disregarded in downstream analyses both by GATK [19] and SAMtools [20]. SNPs were called across the GBS data using GATK.

### 2.4. Population Structure, Linkage Disequilibrium and Genome-Wide Diversity

Quality control (QC) steps, linkage disequilibrium (LD), genome-wide diversity (observed and expected heterozygosity), as well as admixture analyses were performed using TASSEL V5.0 [21]. Variants with MAF < 0.05 and Call Rate < 0.95 were removed. Of the 41,897 SNPs, 256 markers were monomorphic, and 27,375 markers were deleted because of low minor allele frequency (MAF < 0.05). Nei’s distances and pairwise *F*_ST_ among populations were calculated by using the StAMPP package in R [22]. To investigate population structure, R packages including vcfR, poppr, ape, and RColorBrewer were used for K-means clustering and discriminant analysis of principal components (DAPC). The median joining network was created in SplitsTree by using the genetic distance matrix. Relatedness among genome-wide SNPs based on the unadjusted Ajk statistic was evaluated by using TASSEL. The LD between two SNPs was evaluated using the statistics *r*^2^ (Equation (1)) [23]:

(1) $$r^{2}=\frac{(P_{Ab}P_{aB}-P_{AB}P_{aB}){\;}^{2}}{\left(P_{A}P_{B}P_{a}P_{b}\right)}$$

where *P_AB_*, *P_ab_*, *P_Ab_*, and *P_aB_* are the haplotype frequencies and *P_A_*, *P_a_*, *P_B_*, and *P_b_* are the allele frequencies, respectively. The LD decay plot was created in popLDecay [24].

### 2.5. Effective Population Size and Tajima’s D

Effective population size (*Ne*) was predicted using the pairwise LD [3]. The SNeP tool was used for predicting effective population size [25] based on the relatedness among *r*^2^, *Ne*, and *c* (Equation (1) [26]). The corrected *r*^2^ was estimated following Equation (3) [27]:

(2) $$E(r^{2})={\left(1+4N_{e}c\right)}^{-1}$$

(3) $$r_{adj}^{2}=r^{2}-{\left(\beta n\right)}^{-1}$$

where *c* is the recombination rate, n is the number of individuals sampled; *β* = 2 when the gametic phase is known and *β* = 1 if instead the phase is not known. *Ne* during *t* generations ago was predicted (Equation (4)):

(4) $$N_{T\left(t\right)={\left(4ƒ\left(c_{t}\right)\right)}^{-1}\left(E{\left[r_{adj}^{2}|c_{t}\right]}^{-1}-\alpha \right)}$$

where *N_T_* is the effective size *t* generations ago, computed as *t* = (2ƒ(*c_t_*)) − 1 [28]; *c_t_* is the recombination rate defined for a specific physical distance between markers and optimally adjusted with the mapping functions mentioned above; $r_{adj}^{2}$ is the LD value adjusted for sample size; and *α* = {1, 2, 2.2} is a correction for the mutation rates [29].

Tajimas’s D was estimated to investigate demography or signals of selection (Equation (5)) [30].

$$Tajima\_D=\frac{\left(\theta_{\pi }-\theta_{W}\right)}{\sqrt{var(\left(\theta_{\pi }-\theta_{W}\right)}}$$

(5) $$\theta_{W}=\frac{S}{\alpha_{1}}\;\cdot \;\alpha_{1}=\overset{n-1}{\underset{i=1}{\sum }}\frac{1}{i}\;\cdot \;\theta_{\pi }=\frac{\sum i<j^{k_{ij}}}{\left(\frac{n}{2}\right)}$$

where *k_ij_* is the difference between sequence *i* and *j*.

## 3. Results

### 3.1. Genomic Diversity and Linkage Disequilibrium

**Figure 2:**
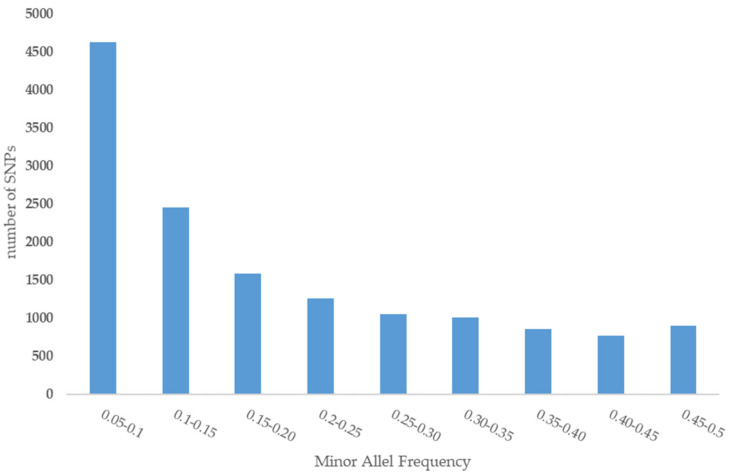
Minor allele frequency of SNPs.

A total of 14,522 SNPs resulted after filtering and were used for final analysis. The largest gap between SNPs (21,088.280 kb) was located on chromosome 11. We note that this is the first SNPs-based report at chromosome-level (Table 1). Before quality control (QC), the average MAF of all SNPs was 0.08, and after filtering, it increased to 0.19 (Figure 2). Average observed heterozygosity in the Iranian central desert dromedaries was estimated at 0.25 ± 0.03.

**Figure 3:**
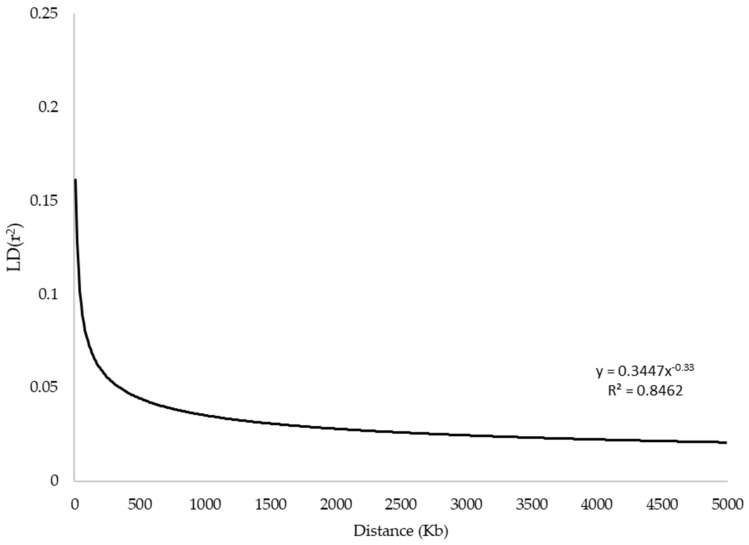
The linkage disequilibrium (LD) decay plot.

Linkage disequilibrium (LD) was estimated for each pairwise combination of SNPs (a total of 242,265 SNP pairs). Average LD (*r*^2^ = 0.089 ± 0.234) was observed at distances shorter than 40 kb, which decreased to 0.019 ± 0.034 in 40–60 kb. The LD decay trend is shown in Figure 3. Ninety-eight percent of total pairwise combinations with *r*^2^ > 0.20 related to 0–40 kb intervals (Table 2).

### 3.2. Cluster Analysis and Genomic Relationship

**Figure 4:**
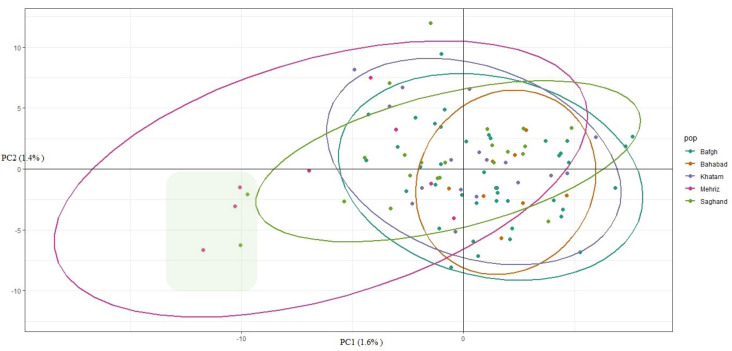
Camels clustered on the basis of principal components analysis using individual genotypes. The highlight box shows five samples (GBS046, GBS047, GBS042, GBS057, and GBS058) which belong to outside of Iran.

**Figure 5:**
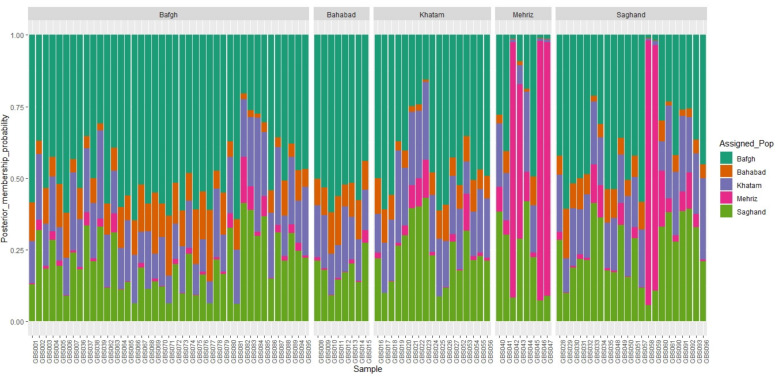
ADMIXTURE plots of 5 dromedary camel populations in the Iranian central desert.

Cluster analysis of the 96 dromedary camels for five regions in the central desert of Iran has been shown in Figure 4. Because of low genetic distance, there were no determined clusters and high admixture among regions (Figure 5). Only 1.6% and 1.4% of the genetic variance was illustrated with PC1 and PC2, respectively, which demonstrated that individuals are relatively homogeneous. Nonetheless, five samples that originate from outside of Iran (Pakistan: GBS046, GBS047, GBS042, GBS057, and GBS058) diverged from the rest (Figure 4 and Figure 5). The pairwise *F*_ST_ statistic among five populations of Iran’s central desert camels was very low (0.002–0.011) (Table 3).

Because of a limited number of bull camels and half-sib calves within herds, there were node clusters visible, although the median of relatedness among camels was very low (A_jk_ = −0.011 ± 0.113).

**Figure 6:**
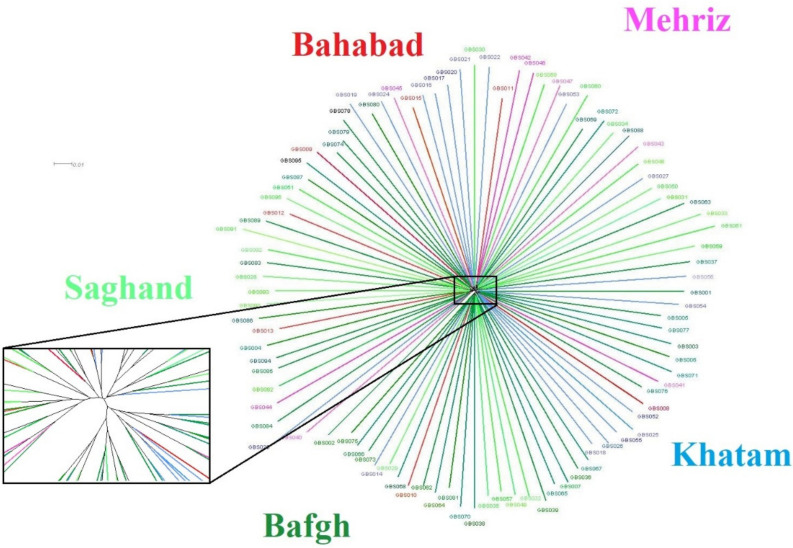
SplitsTree network. Phylogenetic network calculated with SplitsTree using Neighbour-net, with a zoom to visualize the split among five regions of Iran’s central desert.

There is no obvious population structure visible in the Iranian central desert dromedaries, although, in the zoom, it looks as if there was a very low separation into two groups, which, however, do not correspond to any geographical origins nor to any functional separation of the investigated dromedaries in terms of usage for milk or meat production (Figure 6).

### 3.3. Demographic History (Effective Population Size)

**Figure 7:**
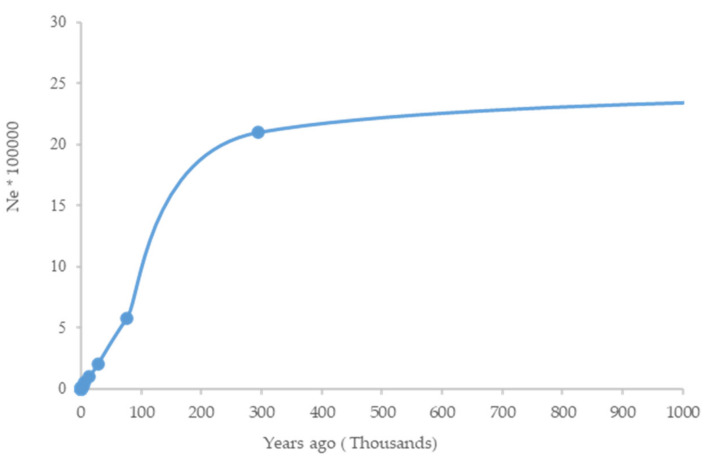
The historical demography of the dromedary camel was examined using SNeP.

Recent Ne (up to 10 generations ago) was estimated as 89 to 487, respectively (Table 4). The trend of Ne shows a great decrease of up to 90% until the last glacial period (LGP; 100,000–20,000 years ago and another bottleneck around 2000–6000 years ago (Figure 7)).

## 4. Discussion

Guo et al., (2020) identified 2,433,732 variants in 366 camels, including the seven domestic *Camelus bactrianus* breeds in East Asia by using the GBS technique [31]. Bahbahani et al., (2019) reported 39,843 SNPs after QC in Sudanese dromedary camels [11]. Al Abri et al., (2017) reported ~80,000 SNPs by using WGS of 9 camels and GBS of 244 dromedary camels [32]. The different numbers of SNP_S_ across different studies can be due to different techniques or the heterozygosity levels in the other camel groups.

Heterozygosity of the Iranian central desert dromedaries was predicted as low (0.25 ± 0.30). Arabian Peninsula and Sudanese dromedaries’ ${\bar{H}}_{O}$ where 0.560 ± 0.003 and 0.347 ± 0.003, respectively [11]. The camels sampled from the central desert of Iran have higher relationships than dromedaries from Sudan (median A_jk_ = − 0.007 ± 0.018) and lower ones than individuals from the Arabian Peninsula (median A_jk_ = 0.347 ± 0.093) [11]. Predicted Tajima’s D (1.28) suggested a bottleneck or balancing selection in dromedary camels in the central desert of Iran. The sudden decrease of camels in Iran could be the cause of a positive Tajima’s D. The number of camels in Iran declined 46% during 1960–1970, however the heterozygosity has been still maintained, most probably by balancing selection resulting in positive Tajima’s D. Balancing selection occurs when the heterozygotes have a higher fitness than the homozygote [33] and conserve genetic polymorphism.

Until recently, LD patterns of many livestock species have been reported. For example, the amounts of LD at 0–30 kb distances in Angus, Charolais, and C beef cattle were 0.29, 0.22, and 0.21, respectively [34]. The mean of *r*^2^ (LD) was 0.155, 0.156, and 0.128 across all chromosomes for three sheep breeds [35]. Corbin et al. (2010) [36] reported *r*^2^ = 0.6 in 5 kb and up to 20 kb in Thoroughbred horses. The average *r*^2^ > 0.2 at 1.0–1.5 Mb intervals was predicted in two pig populations [37]. The mean of LD in Iranian central desert dromedary camels was predicted as very low compared to livestock animals because of high generation interval, lack of artificial selection, and natural reproduction.

Principal component analysis suggested that Sudanese camel populations are almost homogeneous [11]. On the contrary, PCA results distinguished the Chinese Bactrian camels from the Mongolian Bactrian camels very well [31]. The results of PCA, Admixture, and SplitTree tests show that dromedary camels in the central desert of Iran are homogeneous and have a separate genetic background from outside of Iran.

The trend of Ne shows a bottleneck around 2000–6000 years ago in Iranian central desert dromedary camels. This is consistent with a decline in Ne in Old World camels around 4000–5000 years ago and might be related to the domestication scenarios of dromedary and Bactrian camels [2].

## 5. Conclusions

Commercial SNP arrays have been developed for many livestock animals but no SNPchip is available for dromedaries. GBS based on whole-genome sequencing has solved the problem of ascertainment bias [38] as the efficiency of GBS markers is comparable to SNP arrays [39]. GBS could prove highly rewarding for predictions of the relatedness and genetic diversity of dromedaries. This research, the first report of GBS-SNPs assembled by near-chromosomes, suggested dromedary camels in the central desert of Iran are homogeneous and different from outside camels. The decrease of effective population size is a threat for Iran’s dromedary camels.

## Figures and Tables

**Table 1:** Genome-wide summary of SNPs.

Chr	Number of SNPs	Mean MAF	Chr	Number of SNPs	Mean MAF
1	150	0.23	19	1655	0.18
2	412	0.22	20	51	0.27
3	78	0.25	21	305	0.21
4	113	0.21	22	20	0.33
5	125	0.20	23	16	0.21
6	413	0.22	24	84	0.21
7	372	0.22	25	1113	0.19
8	281	0.22	26	51	0.24
9	1829	0.19	27	45	0.19
10	353	0.27	28	30	0.17
11	1442	0.20	29	84	0.25
12	231	0.20	30	101	0.21
13	167	0.24	31	195	0.20
14	1062	0.19	32	22	0.25
15	150	0.25	33	144	0.22
16	378	0.19	34	55	0.16
17	379	0.18	35	376	0.20
18	1301	0.18	36	194	0.19
			X	745	0.18

**Table 2:** Pairwise linkage disequilibrium (*r*^2^) for SNPs at various distance.

Distance	N	Mean	SD	% *r*^2^ > 0.2	% *r*^2^ > 0.3
0–40 kb	7,020,569	0.0899	0.234	98.02	94.50
40–60 kb	613,045	0.0199	0.034	0.15	0.52
60–100 kb	735,773	0.0208	0.039	0.36	0.77
100–250 kb	1,082,002	0.0177	0.030	0.13	0.66
250–500 kb	789,072	0.0198	0.035	0.28	0.76
0.5–1 Mb	650,753	0.0175	0.030	0.14	0.30
1–2 Mb	436,200	0.0181	0.033	0.10	0.28
2–5 Mb	808,181	0.0194	0.0351	0.33	0.59
5–10 Mb	719,173	0.0188	0.033	0.21	0.49
10–20 Mb	809,000	0.017	0.029	0.08	0.45
20–50 Mb	841,882	0.0187	0.031	0.10	0.58
50–92 Mb	204,580	0.0164	0.032	0.11	0.10

**Table 3:** Pairwise F_ST_ among populations of Iran’s central desert camels.

	Bafgh	Bahabad	Khatam	Saghand	Mehriz
**Bafgh**	0	-	-	-	-
**Bahabad**	0.003	0	-	-	-
**Khatam**	0.005	0.004	0	-	-
**Saghand**	0.004	0.002	0.004	0	-
**Mehriz**	0.011	0.010	0.011	0.008	0

**Table 4:** Effective population size in dromedary camel across 113 last generations.

Generations Ago	Ne	Dist.	*r* ^2^	*r*^2^SD
1	89	18,735,020	0.010774	0.015864
2	108	16,440,742	0.010643	0.015069
2	136	14,311,335	0.010036	0.013526
3	155	11,950,567	0.011016	0.016526
4	186	10,407,033	0.010833	0.015233
4	220	9,096,109	0.010719	0.014915
6	282	7,360,504	0.010603	0.015451
7	324	6,387,962	0.010813	0.014915
8	396	5,401,410	0.010624	0.014839
10	487	4,410,026	0.010759	0.015362
13	618	3,561,115	0.010637	0.015982
16	799	2,844,204	0.010428	0.01519
21	1001	2,241,547	0.010652	0.014796
25	1237	1,901,651	0.010217	0.014037
35	1694	1,375,758	0.010399	0.014926
44	2116	1,097,526	0.010477	0.014596
60	2883	811,669	0.010442	0.015188
80	3680	617,213	0.010787	0.015267
113	5344	438785	0.01048	0.01491
